# HMGB1-Dependent Triggering of HIV-1 Replication and Persistence in Dendritic Cells as a Consequence of NK-DC Cross-Talk

**DOI:** 10.1371/journal.pone.0003601

**Published:** 2008-10-31

**Authors:** Héla Saïdi, Marie-Thérèse Melki, Marie-Lise Gougeon

**Affiliations:** Institut Pasteur, Antiviral Immunity, Biotherapy and Vaccine Unit, INSERM U668, Paris, France; New York University School of Medicine, United States of America

## Abstract

**Background:**

HIV-1 has evolved ways to exploit DCs, thereby facilitating viral dissemination and allowing evasion of antiviral immunity. Recently, the fate of DCs has been found to be extremely dependent on the interaction with autologous NK cells, but the mechanisms by which NK-DC interaction controls viral infections remain unclear. Here, we investigate the impact of NK-DC cross-talk on maturation and functions of HIV-infected immature DCs.

**Methodology/Principal Findings:**

Immature DCs were derived from primary monocytes, cultured in the presence of IL-4 and GM-CSF. In some experiments, DCs were infected with R5-HIV-1_BaL_ or X4-HIV-1_NDK_, and viral replication, proviral HIV-DNA and the frequency of infected DCs were measured. Autologous NK cells were sorted and either kept unstimulated in the presence of suboptimal concentration of IL-2, or activated by a combination of PHA and IL-2. The impact of 24 h NK-DC cross-talk on the fate of HIV-1-infected DCs was analyzed. We report that activated NK cells were required for the induction of maturation of DCs, whether uninfected or HIV-1-infected, and this process involved HMGB1. However, the cross-talk between HIV-1-infected DCs and activated NK cells was functionally defective, as demonstrated by the strong impairment of DCs to induce Th1 polarization of naïve CD4 T cells. This was associated with the defective production of IL-12 and IL-18 by infected DCs. Moreover, the crosstalk between activated NK cells and HIV-infected DCs resulted in a dramatic increase in viral replication and proviral DNA expression in DCs. HMGB1, produced both by NK cells and DCs, was found to play a pivotal role in this process, and inhibition of HMGB1 activity by glycyrrhizin, known to bind specifically to HMGB1, or blocking anti-HMGB1 antibodies, abrogated NK-dependent HIV-1 replication in DCs.

**Conclusion:**

These observations provide evidence for the crucial role of NK-DC cross-talk in promoting viral dissemination, and challenge the question of the *in vivo* involvement of HMGB1 in the triggering of HIV-1 replication and replenishment of viral reservoirs in AIDS.

## Introduction

Early stages of HIV-1 infection are associated with local recruitment and activation of important effectors of innate immunity, NK cells and DCs. In the first hours and days of mucosal infection, HIV-1 crosses the epithelial barrier and infects CCR5-expressing DCs, macrophages and T cells in the mucosal tissues to initiate infection [1], [2]. DCs express CD4, CCR5, DC-SIGN [3] and other C-type lectin receptors (CLRs) that facilitate capture and dissemination of HIV-1 [4], [5]. Immature DCs (iDCs) capture HIV-1 through CLRs [6] and captured virus can be internalized and rapidly transmitted to nearby CD4 T cells, in the form of an infectious synapse [7], [8]. DC-T cell conjugates facilitate productive infection in CD4 T cells [9], and dissemination of the infection to the draining lymph nodes and subsequent other lymphoid tissue compartments is ensured by virus-carrying DCs together with infected macrophages and CD4 T cells [10].

Migration of iDC to T cell area of secondary lymphoid tissues after virus uptake is associated to a maturation process, that allows the resulting mature DC (mDC) to prime an antigen-specific response [11]. Recently, the fate of DCs has been found to be extremely dependent on autologous NK cells [12]. NK-iDC interaction results in activation of NK cells that, in turn, induces DC maturation or killing, depending on their respective density [13]–[15]. DC undergoing maturation secrete several cytokines, such as IL-12 and IL-18, that act as potent inducers of NK cell activation and cytotoxicity [16]–[20]. In turn, once activated, NK cells produce IFN-γ and TNF-α, capable of inducing DC maturation. This phenomenon is dependent on the engagement of NKp30 by ligands expressed on iDC [17], [21], and the down-regulation on iDC of HLA-E, the ligand for CD94/NKG2A inhibitory receptor [22]. Another mechanism was proposed suggesting that NK cells, activated by IL-18 released by iDC at the synaptic cleft, secrete HMGB1, which induces DC maturation and protects DCs from lysis [20]. HMGB1 is a nuclear protein that is present in almost all eukaryotic cells, and it functions to stabilize nucleosome formation, and acts as a transcription-factor-like protein that regulates the expression of several genes [23], [24]. HMGB1 is released from necrotic cells, but it can also be secreted by activated macrophages [25] and activated NK cells [20] in response to inflammatory stimuli, and it is one of the main prototypes of the damage-associated molecular pattern molecules (DAMPs) [26]. It was recently discovered to be a crucial cytokine in the immune system, facilitating the trafficking of inflammatory leukocytes, and being critical for DCs to mature, reach the lymph nodes and sustain the proliferation of antigen-specific T cells, and to promote their polarization towards a T-helper 1 phenotype [27], [28].

The mechanisms involved in NK-DC interaction during viral infections are poorly understood. It was recently reported in murine CMV (MCMV) infection that MCMV-infected DCs were capable of activating syngeneic NK cells *in vitro* and also capable of enhancing NK-cell dependent clearance *in vivo* [29], demonstrating the crucial role of NK-DC cross-talk in controlling viral replication. In HIV infection, NK-DC interaction was found defective in HIV-1-infected viremic, but not aviremic patients, characterized by abnormalities in the process of reciprocal NK-DC activation and maturation, as well as a defect in NK-cell elimination of iDCs [30]. We investigated the role of NK-DC cross-talk on maturation, function, and susceptibility to viral replication of HIV-1-infected iDCs. We discovered that maturation of HIV-1-infected DCs could be triggered by activated NK cells, but it was associated with a strong impairment of mature infected DCs to induce Th1 polarization following their cross-talk with NK cells. In addition, the cross-talk between NK cells and HIV-1-infected iDCs resulted in a dramatic increase in viral replication and proviral DNA expression in DCs. This process was mainly triggered by HMGB1, released both by NK cells and DCs, as a consequence of NK-DC cross-talk. This study provides new insights into how HIV ‘hijacks’ DCs to promote efficiently viral dissemination.

## Results

### Activated NK cells induce the maturation of autologous primary immature dendritic cells infected with HIV-1

**Figure 1:**
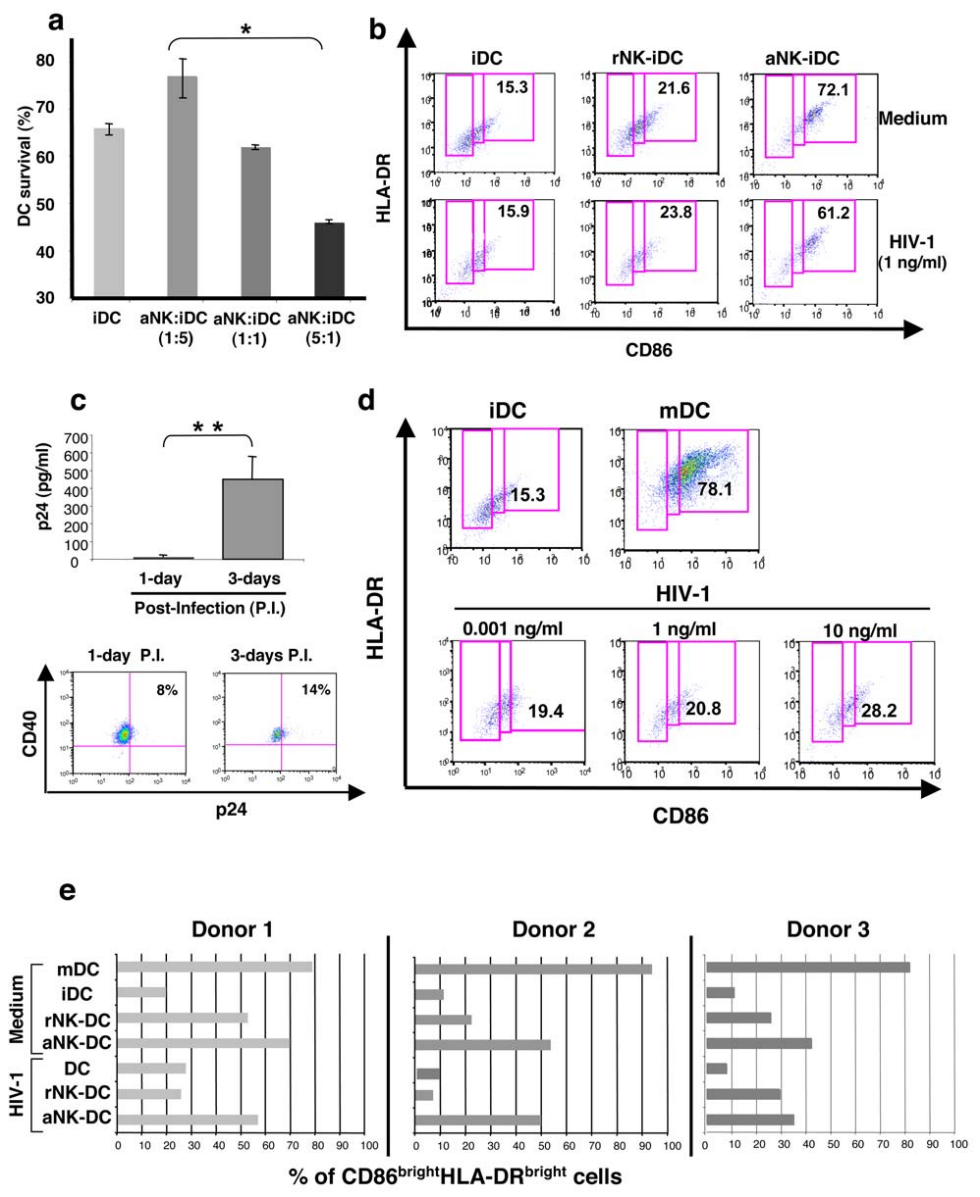
aNK cells induce the maturation of primary immature HIV-1-infected DCs. (a) iDCs, generated from purified CD14^+^ monocytes in the presence of IL-4 and GM-CSF, were cocultured during 24 h with aNK cells at different ratios. DC survival was determined by flow cytometry with the 7-AAD assay. Surviving DCs were identified as 7AAD^−^ CD56^−^ cells. Data represent three independent experiments and values are means±sd. (b) aNK cells induce the maturation of iDCs. Flow cytometry analysis of iDCs, which were either infected with HIV-1_BaL_ (1 ng/ml of p24) for 3 h or uninfected, were incubated with rNK cells or aNK cells at a ratio of 1∶5. Co-staining with HLA-DR and CD86 specific antibodies allowed the identification of mature DCs (CD86^bright^HLA-DR^bright^). Data from a representative experiment out of three independent experiments are shown. (c) The conditions of infection used in this study were those of a productive infection of iDCs, as shown at day 3 by a significant p24 detection in culture supernatant of infected iDCs and intracellular detection by flow cytometry of p24 in DC targeted by CD40 expression. Experiments were performed on DCs from three independent donors, and values are means±sd. (d) HIV-1 infection does not induce by itself the maturation of iDC, as shown by CD86/HLA-DR dual staining of iDCs infected with 0.001 to 10 ng/ml p24 HIV-1_BaL_. The proportion of mDCs induced by LPS (DC0) (78.1% CD86^bright^HLA-DR^bright^) is shown as a positive control. (e) The proportion of mature CD86^bright^HLA-DR^bright^ DCs induced in the indicated cocultures of infected or uninfected iDCs with either rNK or aNK cells are shown. These experiments have been performed on primary cells from a number of donors, and representative data from three of them are shown. When indicated, statistical analyses were made with the non-parametric Mann-Whitney test. * p<0.05, ** p = 0.02.

To investigate the role of NK cells on DC maturation, we generated monocyte-derived DCs from isolated monocytes and cocultured them with NK cells purified from the same donor. NK cells were either resting (rNK) or activated by a combination of PHA and IL-2 (aNK). 24 h of coculture of aNK cells with autologous immature DC (iDC) induced either the survival or apoptosis of iDCs, dependent on NK-DC ratio, consistent with previous reports [14]. Indeed, aNK-DC ratio of 5∶1 induced DC apoptosis, while 1∶5 ratio induced DC survival. (**Fig. 1a**). iDCs survival at a NK-DC ratio of 1∶5 was associated with their maturation, as shown by the increased coexpression of the maturation markers CD86 and HLA-DR (72.1% of CD86^bright^HLA-DR^bright^ DCs were induced by aNK cells compared to 15.3% at baseline) (**Fig. 1b**), a feature of mature DCs. Under the same experimental conditions, rNK cells had a weaker effect on DC maturation, as judged by the proportion of CD86^bright^HLA-DR^bright^ DCs (**Fig. 1b,e**). Following infection of iDC with HIV-1_BaL_, NK-dependent maturation of iDCs was not altered (**Fig. 1b**), under conditions of productive infection of iDCs, measured at day 3 by p24 release in culture supernatant and intracellular staining of iDC for p24 (**Fig. 1c** **)**. Investigating the direct effect of HIV-1 on DC maturation, we found that, at concentrations ranging from 0.001 to 10 ng/ml, HIV-1_BaL_ was unable to increase the expression of the maturation markers CD86 and HLA-DR, in contrast to LPS, used as a positive control as a strong inducer of DC maturation (**Fig. 1b,d**). Data from three representative donors, shown in **Fig. 1e**, confirm the high impact of aNK cells on maturation of iDC after 24 h of coculture, whatever the infected or uninfected status of iDC. These experiments have all been reproduced with X4-HIV-1 and very similar data were obtained (data not shown). From these data, we conclude that productively HIV-1-infected iDCs have maintained a normal susceptibility to maturation induced by NK cells during the NK-DC cross-talk.

### aNK-DC cross-talk triggers HMGB1 expression in both NK cells and DCs

**Figure 2:**
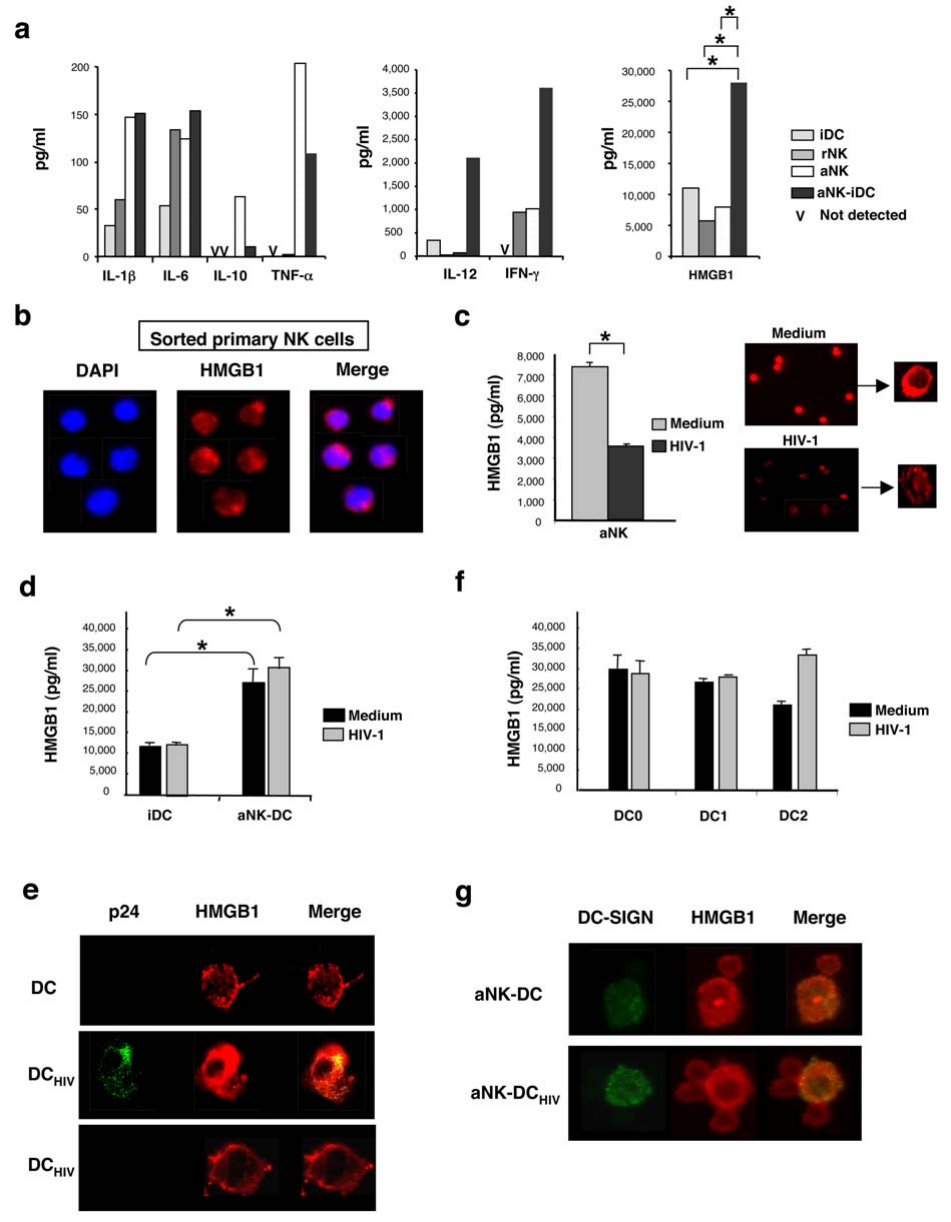
aNK-DC cross-talk triggers HMGB1 expression in both aNK cells and DCs. (a) 24 h cell-free culture supernatants of iDCs, rNK cells, aNK cells (10^6^/ml), or cocultures of aNK cells and iDCs (ratio 1∶5) were tested for cytokine content. MAP technology was used to quantify IL-1β, IL-6, IL-10, TNF-α, IL-12 and IFN-γ, whereas HMGB1 was quantified by ELISA. * p<0.05 (non-parametric Mann-Whitney test). (b) HMGB1 expression was detected by immunofluorescence (in red) in freshly sorted blood NK cells. Counterstaining with DAPI (in blue) showed the nuclear localisation of HMGB1. (c) Incubation of aNK cells with HIV-1 inhibits HMGB1 secretion. Left panel: aNK cells (10^6^ cells/ml) were incubated in medium or with HIV-1_BaL_ (1 ng/ml of p24) for 3 h and tested for HMGB1 production 21 h later. Data represent three independent experiments and values are means±sd. Right panel: immunofluorescence analysis of HMGB1 expression in the same preparations of aNK cells. (d) HMGB1 production during aNK-iDC cross-talk is not inhibited by HIV-1 infection of iDCs. iDCs were incubated for 3 h in medium or with HIV-1_BaL_ (1 ng/ml of p24) and further cocultured for 21 h with aNK cells (aNK∶iDC ratio 1∶5). HMGB1 concentration was then measured in culture supernatants. Data represent the mean±sd of three independent experiments. (e) Immunofluorescence confocal analysis of HMGB1expression in uninfected or HIV-1-infected iDCs. Upper panel: non infected iDCs; middle panel: HIV-1-infected and replicating iDCs, as shown by intracellular p24 staining; lower panel: iDCs incubated with HIV-1 but negative for intracellular p24 expression. (f) Mature DCs were generated by 48 h stimulation of iDCs with LPS (DC0), soluble CD40L (DC1) or LPS+PGE2 (DC2). DC0, DC1 and DC2 were incubated for 3 h in medium or infected with HIV-1_BaL_ (1 ng/ml of p24) and further incubated in medium for 21 h. HMGB1 quantification in culture supernatants was performed. The mean±sd of three independent experiments is shown. (g) Immunofluorescence analysis of HMGB1 expression in conjugates of aNK cells and uninfected (upper panel) or HIV-1-infected DCs (lower panel) in a 24 h coculture. DCs are DC-SIGN^+^ and both aNK cells and DCs express HMGB1 in these conjugates. Pictures from one representative experiment out of three conducted with different primary cell preparations are shown.

In order to identify the molecules involved in aNK-dependent maturation of iDC, we used multianalyte profiling (MAP) to map the key cytokines produced in 24 h culture of iDC, NK cells and aNK∶iDC. iDC released low amounts of IL-1ß, IL-6 and IL-12, and they did not produce IL-10 or TNF-α. Following their coculture with aNK cells, a proinflammatory cytokine profile was induced, with a high increase in IL-12 secretion, significant levels of TNF-α and IFN-γ, both derived from NK cells, and no production of IL-10 (**Fig. 2a**). Interestingly, high levels of HMGB1 were detected in those culture supernatants, originating both from iDC and NK cells, and aNK∶iDC cocultures resulted in a strong enhancement of HMGB1 concentration in culture supernatants (**Fig. 2a**). We confirmed at the single cell level, by confocal microscopy, that NK cells were able to produce HMGB1, detected in the nucleus of freshly isolated NK cells (**Fig. 2b**), and further translocated to the cytoplasm in aNK cells (**Fig. 2c**). Following 3 h incubation with HIV-1_BaL_, aNK cells showed a strong decrease in HMGB1 expression, detected both in culture supernatants and by confocal microscopy (**Fig. 2c**), possibly due to a direct inhibitory effect of HIV virions on NK cell functions through the binding of gp120 [31]. HMGB1 level reached then a level comparable to that of rNK cells (**Fig. 2a**). We checked that NK cells were not able to replicate HIV-1, as shown by the lack of p24 detection in culture supernatant and the lack of intracellular p24 staining (detected by FACS) in NK cells (data not shown). HMGB1 was also secreted by iDCs and, once infected, they still produced comparable amount of the cytokine in culture supernatants (**Fig. 2d**). HMGB1 was mostly detected in the cytoplasm of iDCs, whether infected by HIV-1 or not (**Fig. 2e**), and p24 expression in infected DCs did not alter HMGB1 expression, as shown by dual intracellular staining for p24 and HMGB1 (**Fig. 2e**). When iDCs were cocultured with aNK cells, a strong induction of HMGB1 secretion in culture supernatants was observed (**Fig. 2d**), reaching levels comparable to those produced by mature DCs, i.e. DC0, DC1 and DC2 (**Fig. 2f**). Strikingly, HIV-1 infection of iDC did not affect the amount of HMGB1 produced in NK-DC cocultures (**Fig. 2d**) and in cultures of mature DCs (**Fig. 2f**). Confocal microscopy analysis showed the formation of conjugates between aNK cells and iDCs, which were also observed when aNK cells were cocultured with HIV-1-infected DCs, and both cells expressed HMGB1, whatever the infected status of DCs (**Fig. 2g**). These results demonstrate that HMGB1 is expressed both by NK cells and iDCs during NK-DC cross-talk, and this process is not altered by HIV-1 infection of iDCs.

### aNK-dependent maturation of HIV-1-infected iDCs is mediated by HMGB1 and involves RAGE

**Figure 3:**
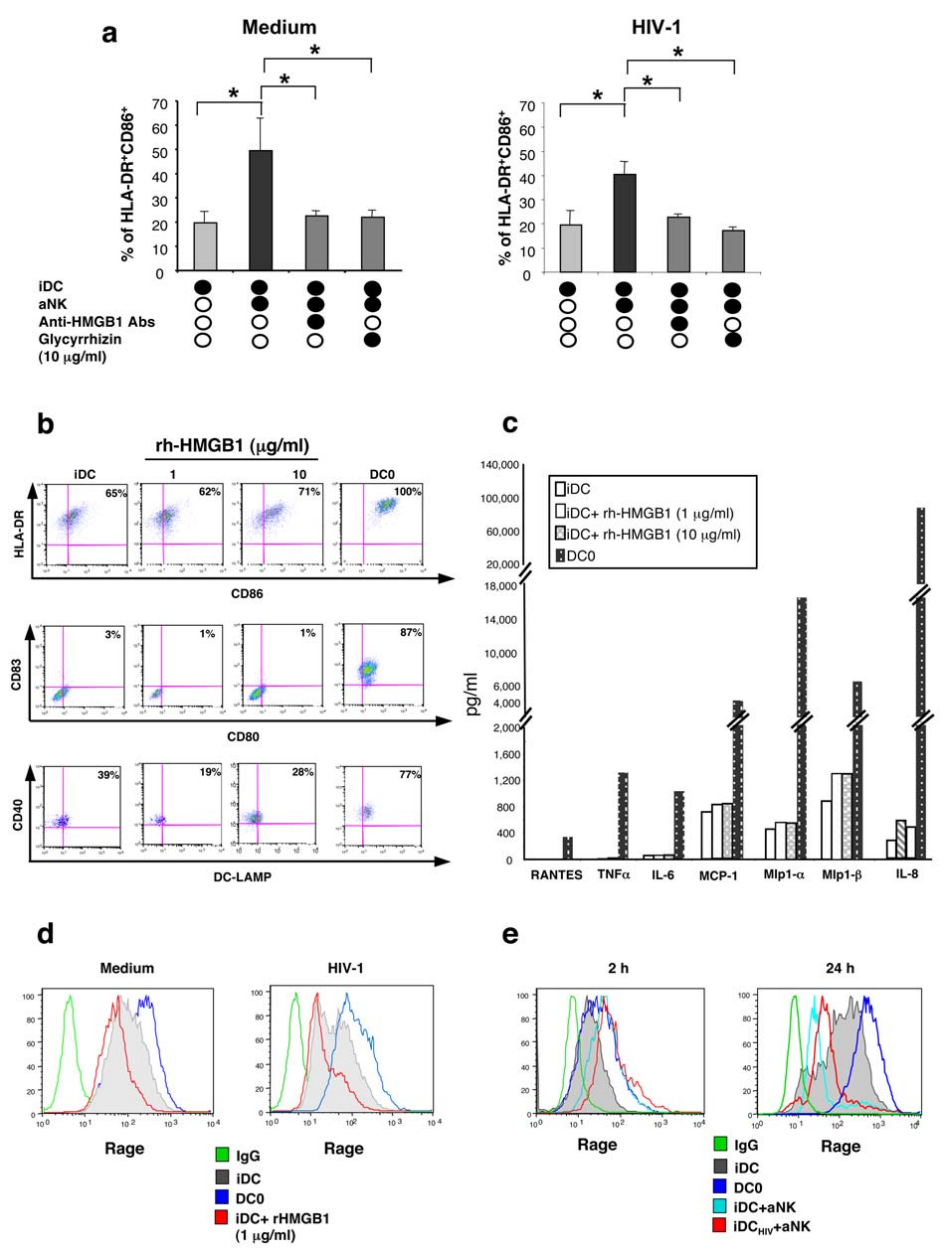
aNK-dependent maturation of HIV-1-infected iDCs is mediated by HMGB1 and involoves RAGE. (a) Left panel: iDCs were cultured for 24 h either alone or with aNK cells, in the presence of blocking anti-HMGB1 antibodies (10 µg/ml) or glycyrrhizin (10 µg/ml). The maturation status of DCs was determined by flow cytometry with CD86 and HLA-DR –specific antibodies. Right panel: same experiment, but performed with HIV-1 infected iDCs. Data represent mean±sd of at least three independent experiments, and statistical comparisons were made with the non-parametric Mann-Whitney test. * p<0.05. (b) iDC (10^6^ cells/ml) were cultured for 48 h with increasing concentrations (1–10 µg/ml) of rh-HMGB1. Cells were then stained with anti-CD86, -HLA-DR, -CD80, -CD83, DC-LAMP and -CD40 antibodies and analysed by flow cytometry. (c) Influence of rh-HMGB1 on cytokine and chemokine production (determined by MAP) by DCs. iDCs (10^6^ cells/ml) were incubated for 48 h in medium or in presence of rh-HMGB1 (1 or 10 µg/ml). As a positive control, iDCs were stimulated with LPS (DC0). (d) Flow cytometry detection of surface expression of RAGE by iDCs, DC0, or iDCs incubated with rh-HMGB1 (1 µg/ml). iDCs were either non infected or infected with HIV-1_BaL_ (1 ng/ml p24 for 3 h). (e) iDC, DC0, uninfected or HIV-1-infected iDC cocultured for 24 h with aNK cells, were incubated with rh-HMGB1 (1 µg/ml) and subsequently stained with anti-RAGE antibodies and analyzed by flow cytometry. NK cells were excluded from the analysis through the co-staining with CD3- and CD56-specific antibodies (CD3^−^CD56^+^).

To determine the possible involvement of HMGB1 in NK-dependent DC maturation, we used glycyrrhizin, known to interact specifically with soluble HMGB1 molecule [32], and anti-HMGB1 antibodies (**Fig. 3a**). These inhibitors, added at the initiation of the 24 h aNK-iDC coculture, reduced the proportions of mature DCs (identified as CD86^bright^HLA-DR^bright^) to the baseline level observed without aNK cells (**Fig. 3a**). Similar effect was obtained with infected DCs (**Fig. 3a**). rh-HMGB1 by itself did not induce phenotypic maturation of iDC, when treated for 24 h with 1 to 10 µg/ml rh-HMGB1, and similar data were obtained at 48 h of culture (**Fig. 3b**). Indeed, while spontaneous maturation of iDCs was observed after 48 h of culture in medium, as shown by the high percentage of CD86^bright^ HLA-DR^bright^ DCs, 10 µg/ml rh-HMGB1 only weakly increased from 65% to 71% the percentage of these cells. Interestingly, rh-HMGB1-treated DCs were not fully mature, as assessed by the lack of expression of CD80, CD83 and the weak expression of DC-lamp, all fully expressed in mDC (DC0) (**Fig. 3b**). However, these partially mature DCs were functionally susceptible to rh-HMGB1 as shown by the increased release of the chemokines, MCP1, MIP-1α, MIP-1β and IL-8 by hr-HMGB1-treated DCs (**Fig. 3c**). HMGB1 receptors include RAGE [33], [34] TLR-2 and TLR-4 [35]. RAGE was the first identified receptor for HMGB1, it is expressed by a variety of immune cells including T cells, monocytes, macrophages and DCs [36], and it is used by maturing DCs for *in vivo* homing to lymph nodes [37]. While TLR-2 and TLR-4 were hardly detected on iDC (not shown), RAGE was fully expressed on DCs, as shown by flow-cytometry, and its expression was even higher on mature DC0 (**Fig. 3d** **)**. Following incubation of iDCs with 1 µg/ml of HMGB1, down-regulation of RAGE was observed, strongly suggesting that this receptor was used by these cells (**Fig. 3d**). Following DC infection with HIV-1_BaL_, no change in RAGE levels was detected on iDC and DC0. Incubation of infected DCs with HMGB1 induced similar down-regulation of RAGE (**Fig. 3d**). The possible involvement of RAGE during NK-DC cross-talk was evaluated with the same approach, comparing RAGE expression on DCs cocultured with aNK cells and DC cultured alone. After 2 h of coculture with aNK cells, DCs showed an up-regulation of RAGE expression, followed by a down-regulation at 24 h (**Fig. 3e**). Very similar observations were made with HIV-1-infected DCs (**Fig. 3e**). Thus, HMGB1 is an important factor for the maturation of both uninfected and HIV-1-infected iDCs during NK-DC cross talk, and it involves RAGE, whose expression on iDC is not altered following their productive infection.

### Impairment of Th1 polarization by HIV-infected DCs as a consequence of a defective NK-DC cross-talk

**Figure 4:**
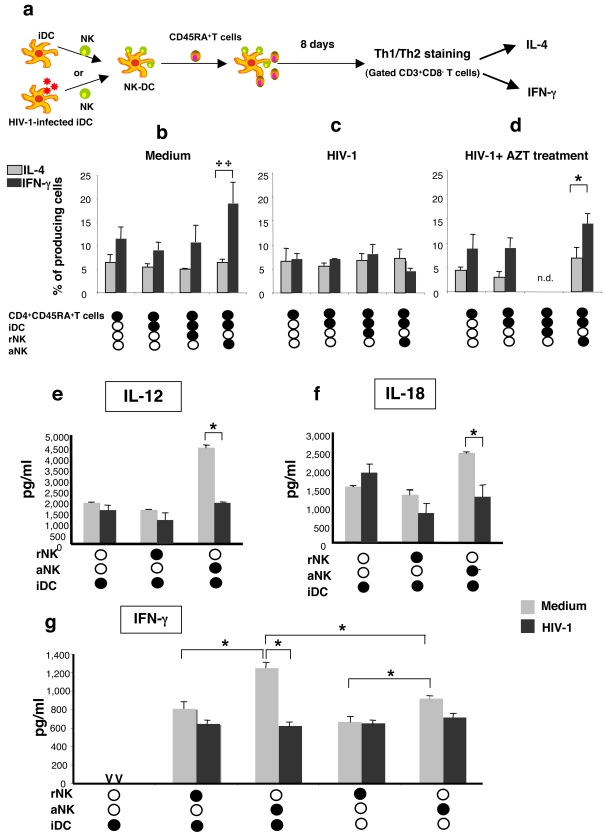
Impairment of NK-triggered Th1 polarization by DCs following HIV-1 infection is associated to altered IL-12 and IL-18 production. (a) Th1 polarization by DCs triggered by NK cells was tested by incubating iDC (10^6^/ml) for 30 mn in the presence of rNK or aNK cells (2×10^5^/ml). Naïve CD4 T cells (10^6^/ml) were added to the cocultures and the frequency of T cells producing IFN-γ or IL-4 was determined by flow cytometry 8 days later. The experiment was performed with either uninfected iDCs (b) iDCs infected with HIV-1_BaL_ (c), or iDC infected with HIV-1_BaL_ in the presence of AZT (1 mM) (d). Culture supernatants of indicated cultures were tested for IL-12 (e), IL-18 (f), and IFN-g (g) content. Data represent the mean±sd of five independent experiments. Statistical comparisons were made with the non-parametric Mann-Whitney test. * p<0.05, **p = 0.03.

It has been shown that the interaction of NK cells with iDCs results in the induction of type-1 polarized DCs that serve as carriers of the NK cell-derived help for the induction of Th1 responses [38]. To assess the capacity of DCs, whether infected or uninfected, to polarize a Th1 response following their cross-talk with aNK cells, naïve CD4^+^CD45RO^−^ T cells were cocultured for 8 days in the presence of DCs and aNK cells, and Th1 polarization was determined by the detection in T cells of the intracellular production of IFN-γ and IL-4, measured by FACS (**Fig. 4a**). Coculture of naïve T cells with iDCs did not increase the proportion of IFN-γ positive T cells, and similar data were obtained in coculture of naïve T cells with iDCs and rNK cells. In contrast, cocultures of naïve T cells with iDC in the presence of aNK cells induced a significant increase of IFN-γ T cell response (**Fig. 4b**), suggesting that aNK∶iDC cross-talk is essential for Th1 polarization. When the same experiment was performed with HIV-1_BaL_-infected DC, no Th1 polarization was observed (**Fig. 4c**). The contribution of HIV-1 replication to the inhibition of Th1 polarization was shown by the addition of AZT, which restored the increased IFN-γ T cell response induced by infected DCs cocultured with aNK cells (**Fig. 4d**). We used AZT at a concentration inhibiting viral replication in these conditions, as assessed by the dosage of p24 antigen in the supernatants (data not shown). IL-12 and IL-18 are critical cytokines produced by DCs and involved in Th1 polarization. We therefore addressed the question of the impact of aNK-DC cross-talk on the release of these cytokines by DCs. We found that aNK-DC cross talk triggers both IL-12 and IL-18 secretion by non infected DCs. Importantly, the production of both cytokines was not detected anymore in cocultures of aNK cells and infected DCs (**Fig. 4e, f**). In addition, the triggering of IFN-γ production by NK cells during aNK-DC cross talk was not detected anymore when the coculture was performed with HIV-1 infected DC (**Fig. 4g**). Thus, the priming of DCs for Th1 polarization occurs during aNK-iDC cross-talk, though the induction of cytokines such as IL-12 and IL-18 released by DCs, and IFN-γ released by NK cells. Following their infection with HIV-1, iDCs cannot be polarized anymore by aNK cells, due to a defective NK-DC cross-talk. Consequently HIV-1 infected DCs are impaired in their capacity to induce Th1 polarization.

### Pivotal role of HMGB1 in NK-DC dependent triggering of HIV-1 replication and persistence in iDCs

**Figure 5:**
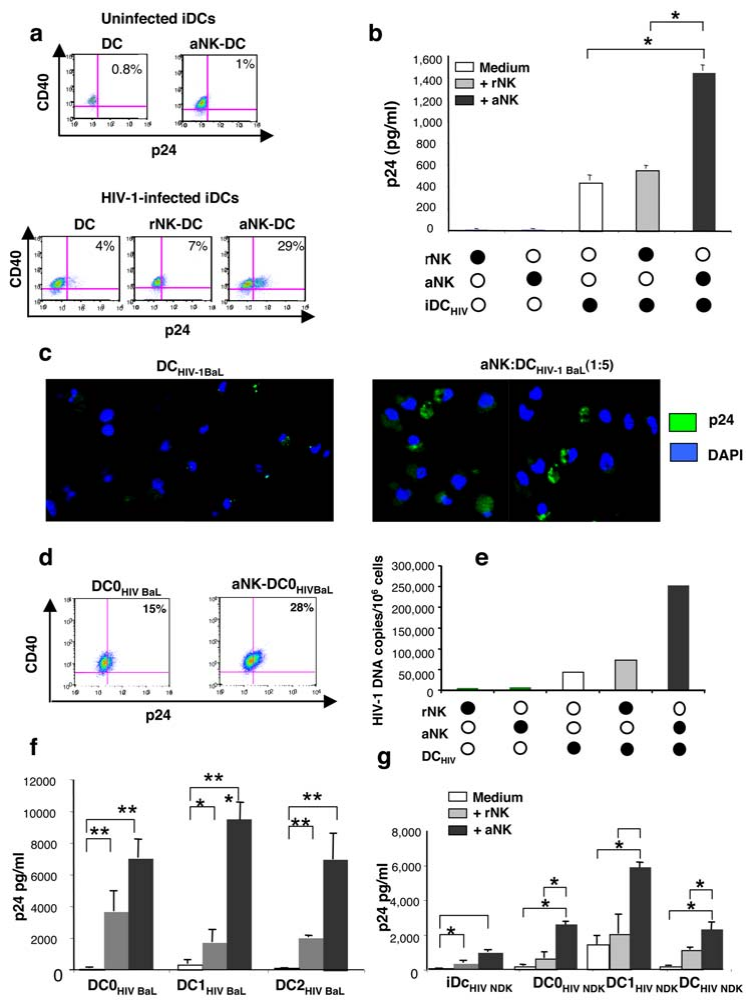
HMGB1-dependent triggering of HIV replication in DC as a consequence of NK-DC cross talk. (a) Flow cytometry analysis of p24 intracellular expression in iDCs (CD40^+^), either uninfected (upper panel) or infected with HIV-1_BaL_ (lower panel) following 3 day-incubation at 10^6^/ml, either alone, or in the presence of rNK or aNK cells (2×10^5^/ml). (b) p24 concentration in culture supernatants of same cultures. Mean±sd of three independent experiments. *p<0.05, non-parametric Mann-Whitney test. (c) Immunofluorescence analysis of intracellular p24 expression in HIV-1-infected iDCs cultured for 3 days either alone or in the presence of aNK cells. Nuclei are stained with DAPI. (d) Flow cytometry intracellular p24 expression in HIV-1-infected DC0 (10^6^/ml) cultured either alone or in the presence of aNK cells for 6 days. (e) HIV-1 proviral DNA levels, determined by light cycler analysis on cells from indicated cultures. One representative experiment out of three conducted with different primary cells preparations is shown. (f) p24 concentration in culture supernatants of mature DCs infected with HIV-1_BaL_ and cultured for 6 days either alone or in the presence of rNK or aNK cells Mean±sd of three independent experiments. Statistical comparisons were made with the non-parametric Mann-Whitney test. * p<0.05, **p = 0.03. (g) p24 concentration in culture supernatants of either iDCs or mature DCs infected with HIV-1_NDK_ and cultured under the same conditions as in (f). Mean±sd of three independent experiments. Statistical comparisons were made with the non-parametric Mann-Whitney test. * p<0.05.

Since we showed that the impairment of Th1 polarization by NK-sensitized HIV-1-infected DCs was dependent on HIV-1 replication (**Fig. 4d**), we tested whether aNK-iDC interaction could trigger HIV-1 replication in DCs. iDCs were infected for 3 h with R5- or X4-HIV-1 (1 ng/ml of p24) and further cultured either alone or in the presence of rNK or aNK for 18 h, and the frequency of DCs with intracellular expression of p24 was determined by flow cytometry. While the percentage of p24^+^ DCs was quite low when HIV-infected iDCs were cultured alone, it significantly increased following their interaction with aNK cells, the p24^+^ DCs representing almost one third of all DCs as compared to only 4% in the absence of NK cells (**Fig. 5a**). Under the same conditions, rNK cells had no effect on HIV replication in iDCs (**Fig. 5a**). aNK-dependent increased HIV replication in infected DCs was confirmed by p24 antigen detection in culture supernatants, and a statistically significant increase of p24 production was detected in cocultures of aNK with HIV-1-iDC as compared to infected iDCs cultured alone or with rNK cells (**Fig. 5b**). The dramatic effect of NK-DC interaction on the frequency of p24-expressing DCs was confirmed by confocal microscopy with p24-specific antibodies. While very rare DCs were stained for intracellular p24 on the day following their infection, a high number of p24^+^ DC were observed after their culture with aNK cells (**Fig. 5c**). Interestingly, the positive influence of aNK cells on HIV replication in iDCs was similarly observed on mature DCs. An increased frequency of p24^+^ DCs, detected by FACS, was found in HIV-1-infected DC0 cocultured during 24 h with aNK cells as compared to DC0 cultured alone (**Fig. 5d**), and p24 detection in culture supernatants from HIV-1-infected mature DC0, DC1 and DC2 cocultured with aNK cells, confirmed the significant stimulating effect of aNK cells on HIV-1 replication in mature DCs. This was observed whether DCs were infected with X4- or R5-HIV-1) (**Fig. 5f and 5g**). Of note, rNK cells had a weak impact on HIV-1 replication in mature infected-DCs (**Fig. 5f and 5g**). We then tested whether aNK cells had an influence on the expression of proviral DNA in iDCs. Data in **Figure 5e** show that a very high increase in the number of HIV-1 proviral DNA copies was detected in cultures of infected iDCs with aNK cells, as compared with that of infected iDCs with rNK cells or infected iDCs alone.

**Figure 6:**
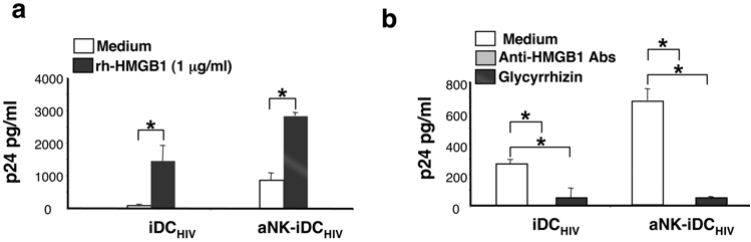
Exogenous rh-HMGB1 triggers HIV-1 replication in iDC. (a) iDC -infected with HIV-1_BaL_ were cultured either alone or in the presence of aNK cells for 3 days. rh-HMGB1 (1 µg/ml) was added in some cultures. HIV replication was measured by p24 quantification in culture supernatant. (b) HIV-1-infected iDC were cultured either alone or in the presence of aNK cells for 3 days. Blocking anti-HMGB1 antibodies (10 µg/ml) or glycyrrhizin (10 µg/ml) were added at culture initiation. HIV replication was measured by p24 quantification in culture supernatant. The mean±sd of three independent experiments is shown. Statistical comparisons were made with the non-parametric Mann-Whitney test. * p<0.05.

Exogenous HMGB1 was recently reported to increase HIV-1 replication in infected monocytic cell lines [39], and to induce *in vitro* the reactivation of HIV-1 in PBMCs from HIV-1-infected patients under antiretroviral therapy [40]. Therefore, we addressed the question of the role of HMGB1 in the NK-dependent triggering of HIV replication in DCs. We found that exogenous rh-HMGB1 had a direct effect on HIV-1_BaL_-infected iDC, enhancing dramatically the production of p24 in culture supernatants (**Fig. 6a**). rh-HMGB1 had also a significant stimulatory effect on p24 production by HIV-1-infected iDC cocultured with a NK cells (**Fig. 6a**). To investigate the influence of HMGB1 in the triggering of HIV-1 replication in infected-iDC-aNK cocultures, HMGB1-specific neutralizing antibodies or glycyrrhizin were added to these cocultures and p24 production was measured in the supernatant. Both HMGB1 inhibitors abrogated HIV-1 production by infected DC cocultured with aNK cells or cultured alone (**Fig. 6b**). These results indicate that exogenous HMGB1 is able to trigger HIV-1 replication by infected iDC. They also indicate that aNK cell-dependent stimulation of HIV-1 replication in iDCs is mediated by HMGB1.

## Discussion

Although direct infection of DCs is less efficient than infection of CD4^+^ T cells [41], [42] an increasing amount of evidence indicates that long-term HIV transmission that is mediated by DCs depends on viral production by the DCs [43]–[45], and HIV-infected DCs *in vivo* might function as viral reservoirs during migration to the lymphoid tissues, thereby helping to spread viral infection. Here we have shown for the first time that activated NK cells stimulate HIV-1 replication in DCs and thus might contribute to the establishment of viral reservoirs in these cells. We demonstrated the NK-cell activating capacity of HIV-1-infected iDCs and the crucial involvement of HMGB1, produced during aNK-iDC cross-talk, in the stimulation of HIV-1 replication and proviral DNA expression in DCs. We also showed a strong impairment of mature infected DCs to induce Th1 polarization following their cross-talk with NK cells. Our observations reveal novel HIV strategies to promote efficiently viral dissemination and escape the immune system.

Interaction of NK cells with autologous iDCs results in reciprocal activation, and this interaction seems crucial in the initiation/amplification of the early phases of an immune response, before T cells are generated [11]. NK cells trigger iDCs to mature, and this occurs through an HMGB1-dependent mechanism [20]. NK-dependent maturation of iDCs was reported to involve a functional polarization of DCs, with increase in intracellular free Ca^2+^ concentration, cytoskeleton rearrangement, accumulation of secretory lysosomes at the NK/DC synapse, and regulated expression of IL-18 toward the interacting NK cells. In turn, NK cells secrete large amounts of HMGB1, which induces maturation of DCs [20]. Here, we confirm the involvement of HMGB1 in NK-dependent DC maturation during NK-DC contact, as shown by the inhibitory effect of anti-HMGB1 antibodies or glycyrrhizin, known to interact specifically with HMGB1 [32]. Confocal microscopy analyses and HMGB1 detection in cell-free culture supernatants demonstrated that HMGB1 was not only expressed and secreted by primary NK cells, as reported [20], but it was also produced by isolated DCs, the level of HMGB1 release being linked to their maturation stage. An extremely high level of HMGB1 was detected when iDCs where put in contact with aNK cells, similar to the one released by mature DCs. Interestingly, confocal microscopy analysis of NK-DC conjugates showed that both cells expressed the cytokine. HMGB1 receptor, RAGE, was found rapidly induced following DC interaction with aNK cells, and was further down-regulated, compatible with the implication of HMGB1 in NK-dependent DC maturation. In addition to contributing to DC maturation, HMGB1 has been shown to act as a chemoattractant on iDCs [46], and to be also required for migration of mature DCs in response to CCR7 and CCR4 ligands [47], both activities being mediated by RAGE [46], [47]. Thus HMGB1 acts as an alarmin, having activating and chemotactic effects on DCs, and stimulating then the migration of DCs from inflamed tissues to the draining lymph nodes [46]. These properties of HMGB1 have to be taken into consideration in the context of an uncontrolled viral infection, such as that induced by HIV.

Productive infection of iDCs with R5 or X4 strains of HIV-1 preserved NK-dependent phenotypic DC maturation, as shown by the frequency of CD86^bright^HLA-DR^bright^ DCs, while HIV itself didn't induce DC maturation in the range of p24 concentrations used (0.001 to 10 ng/ml). However, the consequence of aNK-DC interaction was a significant enhancement of HIV-1 infection in iDCs. This was shown by several means, indicating an increased frequency of p24^+^ DC, associated with a significant enhancement of p24 release in NK-DC culture supernatant, and this was confirmed by immunofluorescence at the single cell level. Moreover, NK-DC cross-talk resulted in a dramatic increase in HIV-1 DNA expression in DCs. Considering the crucial role of HMGB1 during the reciprocal activation of DCs and NK cells, we evaluated its contribution to the triggering of HIV-1 replication in iDCs with blocking anti-HMGB1 antibodies or glycyrrhizin. The strong blocking effect of these inhibitors on p24 release indicates the involvement of HMGB1 in the process. It is noteworthy that both inhibitors also decreased significantly HIV-1 replication in 24 h cultures of infected iDCs, in the absence of NK cells. This is likely due to the spontaneous release of HMGB1 by iDC, shown here and previously reported [47], which was preserved following their infection with HIV-1. These observations reveal a pivotal role for HMGB1 in controlling HIV-1 replication in DCs. As a corollary, we demonstrated that rh-HMGB1 significantly increased p24 release in culture supernatants of infected DCs and of aNK-infected DC cocultures. These data may have important implications in the understanding of HIV pathogenesis, since plasma HMGB1 levels were found elevated in chronically HIV-1-infected patients, with the highest concentrations in patients with clinical complications [48]. Moreover, exogenous HMGB1 was reported to induce *in vitro* the reactivation of HIV-1 in PBMCs from HIV-1-infected patients under antiretroviral therapy [40].

Secreted HMGB1 is necessary for proliferation, survival, and polarization of naïve CD4 T cells after activation by allogeneic DCs, and these effects involve RAGE expressed by DCs [49]. Here, we show that, in syngeneic conditions, HMGB1 was not able by itself to induce Th1 polarization. Indeed, no Th1 response was induced in the presence of HIV-1-infected DCs, though they continued to produce normal levels of HMGB1, while being inhibited in the release of IL-12 and IL-18. Recent studies highlighted the essential role of NK cells in the modulation of Th1 polarization, suggesting that they trigger IL-12 and IL-18 release by DCs, promoting the production of IFN-γ by NK cells that in turn trigger the differentiation of T cells towards Th1 cells [50], [51]. The essential role of IL-12 and IL-18 on Th1 differentiation is confirmed here, since the defect of HIV-1 infected DCs to produce increased amount of IL-12 and IL-18 in response to NK cell activation was associated with a defective Th1 polarization. This defect was directly linked to HIV-1 replication in DCs, as shown by the positive effect of the HIV inhibitor AZT. These observations suggest that some of the functional alterations reported in DCs from HIV-infected patients [52], [53], such as a decreased secretion of several cytokines, including IL-12, and an impaired ability to prime autologous CD4 T cells, may be linked to a defective NK-DC cross-talk, as suggested recently [30]. Alteration of NK∶DC cross may also lead to a defect in NK-cell elimination of iDCs, as reported in chronically HIV-infected patients [30] and associated with the resistance of HIV-infected DCs to apoptosis induced by NK cells (Melki MT, Saïdi H, Gougeon ML, submitted to publication).

In summary, we have shown that activation of HIV-1 replication and the possible establishment of viral reservoirs in HIV-1-infected DCs is dependent on a cross-talk between aNK cells and autologous DCs. We have identified the pivotal role of HMGB1 in this process, produced both by NK cells and DCs during their cross-talk, and showed that NK-dependent triggering of HIV replication in DCs is completely abrogated by Glycyrrhizin, which binds specifically to HMGB1, or blocking anti-HMGB1 antibodies. In addition, our data indicate a strong impairment of HIV-1-infected DCs to induce Th1 polarization following their cross-talk with NK cells. These observations provide evidence of the crucial role of NK-DC cross-talk in promoting viral dissemination, and challenges the question of the *in vivo* involvement of HMGB1 in the triggering of viral replication and replenishment of viral reservoirs.

## Methods

### Isolation and separation of primary cells

Peripheral blood mononuclear cells (PBMCs) were separated from the blood of healthy donors on a Ficoll-Hypaque density gradient. PBMC were obtained through the French blood bank (Etablissement Français du Sang, Paris, France) in the setting of EFS-Institut Pasteur Convention. A written agreement was obtained for each donor to use the cells for clinical research according to French laws. Our study was approved by IRB, external (Etablissement Français du Sang Board) as required by French law and internal (Biomedical Research Committee Board, Institut Pasteur) as required by Pasteur Institute.

We isolated CD14^+^ monocytes from PBMCs by positive selection using CD14-specific immunomagnetic beads (Miltenyi Biotech, Auburn, CA). To generate iDCs, purified CD14^+^ monocytes were cultured for 5 days (1×10^6^ cells/ml) in RPMI 1640 medium supplemented with 2 mM glutamine, 10% FCS, penicillin (100 U/ml) and streptomycin (100 µg/ml), in the presence of 10 ng/ml of recombinant human (rhu) GM-CSF and 10 ng/ml rhIL-4 (Peprotech INC, Rockyhill, USA) as described [54]. Culture medium was replaced every 2 days. NK cells were isolated by negative selection from PBMCs depleted of monocytes using a depletion cocktail of antibodies directed to CD3, CD4, CD14, CD19, CD20, CD36, CD123, CD66b, Glycophorin A (StemCell Technologies). The NK cell content of the enriched fraction, determined by flow cytometry (FACScalibur, Becton Dickinson) as CD3^−^CD56^+^ cells with FITC-conjugated anti-CD3 and APC-conjugated anti-CD56 antibodies, ranged from 85 to 95% in the different experiments. Contamination with myeloid cells, evaluated with FITC-conjugated anti-CD14 antibodies was consistently less than 1%. Naïve CD4 T cells (CD4^+^CD45RA^+^) were isolated from PBMCs by positive selection, using CD4- and CD45RA-specific immunomagnetic beads (Miltenyi Biotech, Auburn, CA). Cell purity of isolated naïve CD4 T cells was routinely more than 90%.

### Activation and infection of NK cells

Purified NK cells were cultured at 10^6^ cells/ml either in the presence of suboptimal concentration of IL-2 (100 ng/ml) (Peprotech) to maintain them alive (referred as rNK) or were activated by a combination of PHA (10 µg/ml) (Sigma) and IL-2 (10 µg/ml) (referred as aNK cells). In some experiments, aNK cells (10^6^ cells/ml) were incubated during 3 h in the presence of HIV-1 (1 ng/ml p24) and further cultured for 21 h. Under those conditions, no productive infection could be observed. Culture supernatants were then tested for cytokine and chemokine detection (see below).

### Maturation and phenotypic analysis of DCs

After 6 days of culture in the presence of IL-4 and GM-CSF, iDCs (10^6^ cells/ml) were either non stimulated, or stimulated during 48 h with 10 µg/ml LPS (E. Coli serotype 026-B6, Sigma-Aldrich) to obtain DC0 cells, or 500 ng/ml of trimeric CD40L (Sigma-Aldrich) to obtain DC1 cells, or 10 µg/ml of LPS and 1 µg/ml PGE_2_ (Sigma-Aldrich) to obtain DC2 cells. Phenotypic analysis of DCs and characterization of their maturation stage was performed by flow cytometry. DCs were stained for 20 min at 4°C with antibodies specific for CD80, CD83, CD86, HLA-DR, CD40, DC-LAMP or DC-SIGN (all antibodies from BD Biosciences, San Jose, CA) diluted in 100 µl of PBS/10% FCS/0.1% NaN^3^. In some experiments, antibodies specific for HMGB1-receptor, RAGE (Abcam, Cambridge, UK), was used to stain DCs. After two washings, cells were fixed in 1% PFA, immediately acquired on a FACScalibur (Becton Dickinson) and analyzed with Flow Jo software.

### Infection of DCs with HIV-1

Virus stock preparation was prepared by amplification of R5-HIV-1_BaL_ and X4- HIV-1_NDK_ on MDM and PHA+ IL-2 activated PBMC from healthy donors, respectively. Viral stock was then clarified by centrifugation prior to determination of HIV-1 p24 concentration. iDCs were plated in 96-well culture plates at 200,000 cells/well and incubated for 3 hours at 37°C in a 5% CO_2_ atmosphere with HIV-1 at various concentrations (0.001 to 10 ng p24/ml). Cells were harvested, washed four times with media containing 10% FCS and, when indicated, rNK or aNK cells were added at a NK∶DC ratio of 1∶5, unless otherwise indicated. NK-DC cocultures lasted 24 h before analysis of the maturation stage of DCs and/or quantification of viral production. In some experiments (**Fig. 6**), HIV-1 infected iDCs were incubated alone or with aNK cells for 3 days, in the presence of rh-HMGB1 (1 µg/ml) (R&DSystems), rabbit anti-HMGB1 Abs (10 µg/ml) (Abcam, Cambridge, UK) or Glycyrrhizin (10 µg/ml).

### Quantification of HIV-1 viral production, proviral load and of the frequency of infected DCs

We determined the concentration of HIV-1 in the supernatant of infected cell cultures by measuring the amount of p24 protein by ELISA (Ingen, Belgium). We extracted DNA from cells using the GIAamp DNA Blood Mini Kit (Qiagen, Basel, Switzerland) and quantified HIV-1 proviral load by RT-PCR as described previously [55]. The frequency of HIV-1-infected cells was determined by flow cytometry to detect intracellular p24 molecule. Cells were surface stained with antibodies specific for CD40 (BD Biosciences, San Jose, CA) to target DC and intracellular stained with p24-specific antibodies (Coulter). Stained cells were fixed in 1% PFA, immediately acquired on a FACScalibur (Becton Dickinson) and analyzed with FlowJo software. In some experiments infected DCs were imaged, after immunofluorescence, by laser scanning confocal microscopy.

### Cocultures of iDCs with NK cells

rNK or aNK were cocultured during 24 h with iDCs or mDCs at a ratio of 1∶5 (2×10^5^ NK+10×10^5^ DC/ml), unless otherwise indicated. DC survival was determined with the 7-AAD assay, as described previously [56]. Briefly, cultured cells were stained with 20 µg/mL nuclear dye 7-amino-actinomycin D (7-AAD; St. Quentin-Fallavier, Sigma-Aldrich) for 30 minutes at 4°C, and co-stained with CD56-specific antibody (BD Biosciences, San jose, CA). Surviving DC were identified as CD56^−^ 7-AAD^−^ cells. When phenotypic characterization of DCs was performed in NK-DC cocultures, NK cells were always excluded from the FACS analysis through their staining with CD56-specific antibodies.

### Measurement of cytokine and chemokine production

Cell-free culture supernatants were prepared by incubating for 24 h iDCs at 10^6^ cells/ml, rNK or aNK cells at 2.10^5^ cells/ml or aNK and iDC cells at the ration of 1∶5. Chemokines and cytokines were measured by Luminex (24 plex kits; Biosource) following the manufacturer's instructions. In brief, 50 µl of supernatant or standard was incubated with antibody-linked beads for 2 h, washed twice with wash solution, and incubated for 1 h with biotinylated secondary antibodies. A final incubation of 30 min with streptavidin-PE preceded the acquisition on the Luminex 100IS. At least 100 events were acquired for each analyte. Values above or below the standard curves were replaced by the lowest or the highest concentrations measured. Quantification of HMGB1in cell free culture supernatants was performed with an ELISA kit (IBL, Hamburg). In experiments testing Th1 polarization of naïve CD4 T cells by NK-triggered DCs (Figure 4), quantification of IL-12, IFN-γ and IL-18 in culture supernatants was performed with ELISA kits (IL-12 and IFN-γ kits from R&D Systems, IL-18 kit from MBL).

### Th1 polarization assay

Naïve CD4 T cells (10^6^/ml) were cocultured for 8 days in the presence of uninfected or HIV-1-infected iDC (10^6^/ml) and resting or activated NK cells (2×10^5^/ml) and tested for Th1 polarization by flow cytometry, as previously reported [57]. Briefly, brefeldine A (10 µg/ml) (Sigma Aldrich) was added during the last 16 h of the culture to inhibit protein secretion. Surface staining was performed with PerCP-conjugated CD8 antibodies and FITC-conjugated CD3 antibodies (BD Biosciences, San Jose, CA), followed by cell fixation for 15 minutes at 4°C with 1% PFA and permeabilization with saponin buffer (PBS-BSA 0.2%-NaN^3^ 0.01%- saponin 0.5%), and intracellular staining was performed with APC-conjugated IFNγ ~- or IL-4- specific antibodies (BD Biosciences, San Jose, CA). Stained cells were immediately acquired on a FACScalibur (Becton Dickinson) and analyzed with Flow Jo software. In order to analyze the influence of HIV-1 replication on Th1 polarization, AZT 1 mM was added at the initiation of the culture of naïve CD4 T cells incubated alone, or in the presence of HIV-1-infected iDCs+/−rNK or aNK cells. AZT was left until the end of the coculture. HIV-1-infection of iDCs was performed as described above, in the absence of AZT.

### Stastitical analyses

Statistical analyses were made with the non-parametric Mann-Whitney test. The *P* value of significant differences is reported. Plotted data represent mean±standard deviation (s.d.).
